# CBFB-MYH11 Fusion Sequesters RUNX1 in Cytoplasm to Prevent DNMT3A Recruitment to Target Genes in AML

**DOI:** 10.3389/fcell.2021.675424

**Published:** 2021-07-15

**Authors:** Peng Liu, Jin-Pin Liu, Si-Jia Sun, Yun Gao, Yingjie Ai, Xiufei Chen, Yiping Sun, Mengyu Zhou, Yun Liu, Yue Xiong, Hai-Xin Yuan

**Affiliations:** ^1^The Fifth People’s Hospital of Shanghai and the Molecular and Cell Biology Research Lab of the Institutes of Biomedical Sciences, Fudan University, Shanghai, China; ^2^Department of Gastroenterology and Hepatology, Zhongshan Hospital of Fudan University, Shanghai, China; ^3^Ministry of Education (MOE) Key Laboratory of Metabolism and Molecular Medicine, Department of Biochemistry and Molecular Biology, School of Basic Medical Sciences, Fudan University, Shanghai, China; ^4^Cullgen Inc., San Diego, CA, United States

**Keywords:** DNA methylation, CBFB-MYH11, DNMT3A, mutually exclusive mutation, RUNX1

## Abstract

A growing number of human diseases have been found to be associated with aberrant DNA methylation, including cancer. Mutations targeting genes encoding DNA methyltransferase (DNMT), TET family of DNA demethylases, and isocitrate dehydrogenase (IDH1, IDH2) that produce TET inhibitory metabolite, 2-hyoxyglutarate (2-HG), are found in more than half of acute myeloid leukemia (AML). To gain new insights into the regulation of DNA de/methylation and consequence of its alteration in cancer development, we searched for genes which are mutated in a manner that is linked with gene mutations involved in DNA de/methylation in multiple cancer types. We found that recurrent CBFB-MYH11 fusions, which result in the expression of fusion protein comprising core-binding factor β (CBFB) and myosin heavy chain 11 (MYH11) and are found in 6∼8% of AML patients, occur mutually exclusively with DNMT3A mutations. Tumors bearing CBFB-MYH11 fusion show DNA hypomethylation patterns similar to those with loss-of-function mutation of DNMT3A. Expression of CBFB-MYH11 fusion or inhibition of DNMT3A similarly impairs the methylation and expression of target genes of Runt related transcription factor 1 (RUNX1), a functional partner of CBFB. We demonstrate that RUNX1 directly interacts with DNMT3A and that CBFB-MYH11 fusion protein sequesters RUNX1 in the cytoplasm, thereby preventing RUNX1 from interacting with and recruiting DNMT3A to its target genes. Our results identify a novel regulation of DNA methylation and provide a molecular basis how CBFB-MYH11 fusion contributes to leukemogenesis.

## Introduction

Epigenetic modification plays critical roles in many cellular processes, such as development, differentiation and hemostasis, and, when dysregulated, leads to disease states including cancer. Reversible DNA methylation is one of the major epigenetic modifications that can activate or repress gene expression (Jones, 2012; Yang et al., 2014). Numerous studies have demonstrated altered DNA methylation pattern in tumors compared to normal tissues in many different types of cancers (Galm et al., 2004; Compare et al., 2011; Hinoue et al., 2012; Hon et al., 2012; Shen et al., 2013; Fotouhi et al., 2014; Wedge et al., 2017). Frequent mutations have been found targeting the enzymes involved in both DNA methylation (DNA methyltransferase, predominantly DNMT3A) (Yang et al., 2015) and DNA demethylation [the TET family α-KG/Fe(II)-dependent DNA dioxygenases, mostly TET2] (Rasmussen and Helin, 2016; Lio et al., 2019). In addition, frequent mutations are also found in the genes involved in the regulation of DNA de/methylation function. For example, mutations targeting metabolic enzymes isocitrate dehydrogenase (IDH1, IDH2) produce oncometabolite, 2-hyoxyglutarate (2-HG), that acts an antagonist of α-KG and competitively inhibits TET enzymes (Figueroa et al., 2010; Xu et al., 2011). In addition, mutations targeting transcriptional factor WT1 disrupt its binding with TET2 and impair its recruitment of TET2 in gene regulation (Rampal et al., 2014; Wang et al., 2015).

About 25% of AML sustain mutations targeting genes involved in DNA methylation, mostly DNMT3A (Cancer Genome Atlas Research Network et al., 2013; Brunetti et al., 2017). DNMT3A mutations occur early during leukemogenesis, suggesting an initiating role. DNMT3A mutations are associated with poor prognosis in AML patients (Shivarov et al., 2013). Agents targeting DNA methylation, such as FDA-approved DNA hypomethylating drugs decitabine and azacytidine, are frequently used in treating different types of human cancer, including AML (Derissen et al., 2013). Ablation of *Dnmt3a* in murine hematopoietic stem cells (HSCs) confers a selective advantage for stem cell expansion and maintains their stemness (Challen et al., 2011), and predisposes to multiple types of hematological disease (Celik et al., 2015; Mayle et al., 2015). DNMT3A mutations are associated with global hypomethylation patterns and alteration of the expression of genes important for differentiation, such as HOX genes (Yan et al., 2011; Hajkova et al., 2012; Jeong et al., 2014; Qu et al., 2014), which have an important role in normal hematopoiesis (Alharbi et al., 2013). Whether additional oncogenic events also target DNA methylation process to contribute AML is less well understood.

A frequent cytogenetic abnormality found in AML is chromosome 16 inversion, inv(16), which results in the production of a fusion protein comprising most of core-binding factor-β (CBFB, residues 1–165 of a total 187 residues) and myosin heavy chain 11 (MYH11) (Liu et al., 1993). CBFB-MYH11 fusion occurs in 12% of pediatric and 7% of adult AML (Grimwade et al., 2010; Bolouri et al., 2018) and defines a distinct subtype of AML. Although AML patients bearing CBFB-MYH11 fusion have a relatively good prognosis compared to other AML subtypes, nearly half of the patients become refractory to or relapse after chemotherapy (Borthakur et al., 2008). CBFB is the major partner of evolutionary conserved RUNX transcription factors that play important roles in development by regulating cell proliferation, differentiation and apoptosis (Ito et al., 2015). Heterozygous mice with knock-in of CBFB-MYH11 fusion are defective in hematopoiesis and embryogenesis (Castilla et al., 1996) and are phenotypically similar to mice lacking CBFB or RUNX1 (Okuda et al., 1996; Sasaki et al., 1996; Wang et al., 1996; Niki et al., 1997). These genetic studies suggest that CBFB-MYH11 acts as a dominant repressor of RUNX1 and CBFB during hematopoiesis.

How MYH11 fusion impacts the function of CBFB, a non-DNA-binding regulatory subunit, and the transcriptional activity of RUNX1 are not fully understood. CBFB-MYH11 fusion is predominantly localized in the cytoplasm (Adya et al., 1998; Kanno et al., 1998; Tanaka et al., 1998), although partial localization in the nucleus was also reported (Durst et al., 2003; Mandoli et al., 2014). A plausible model is that CBFB-MYH11 fusion protein sequesters RUNX1 in the cytoplasm, thereby preventing the transcription activity of RUNX1.

In this study, we identified the recurrent CBFB-MYH11 fusions showed significant mutually exclusively pattern with DNMT3A mutations in AML, indicating a novle role of CBFB-MYH11 mutation in epigenetic control. We further demonstrate the underlying mechanism that CBFB-MYH11 fusion protein sequesters RUNX1 in the cytoplasm, thereby preventing RUNX1 from interacting with and recruiting DNMT3A to its target genes. These findings provide an explanation on how CBFB-MYH11 fusion may function in promoting leukemogenesis and reveal a novel regulation of DNA methylation.

## Materials and Methods

### Cell Culture and Transfection

HEK293T, U2OS and K562 cells were purchased from the American Type Culture Collection (ATCC). HEK293T cells were cultured in Dulbecco’s modified Eagle’s medium (GIBCO). U2OS and K562 cells were cultured in RPMI-1640 medium (Invitrogen). All the cell medium mentioned above were supplemented with 10% fetal bovine serum (Gibco) and 50 μg/ml of penicillin/streptomycin. Plasmid transfection was carried out by using polyethylenimine or lipofectamine 2000 (Invitrogen) according to the manufacturer’s instruction.

### Stable Cell Line Establish/Infection

To establish K562 stable cell lines with RUNX1 or DNMT3A knockdown, shRNA oligos targeting RUNX1 or DNMT3A were custom-synthesized, annealed, and inserted into the pLKO.1 plasmid. The shRNA sequences targeting RUNX1 or DNMT3A were listed in Supplementary Table 2. Lentiviruses carrying indicated shRNA were produced in HEK293T cells by co-transfecting pLKO.1 plasmid with packaging plasmids psPAX2 and pMD2.G. After 36 h transfection, lentiviral supernatant was harvested and filtered with 0.45-μm filters. K562 cells were infected with filtered lentiviral media with the addition of polybrene (8 μg/ml). Stable cell pools were obtained after selection with 0.5 μg/ml of hygromycin for 5 days.

To generate K562 stable cells with overexpression of CBFB-MYH11, CBFB-MYH11-MT or DNMT3A, lentiviruses carrying pLVX-vector, pLVX-Flag-CBFB-MYH1, pLVX-Flag-CBFB-MY11-MT or pLVX-Flag-DNMT3A were produced and K562 cells were infected as described above.

### Co-immunoprecipitation and Western Blotting

Cells were washed with ice-cold PBS and then lysed in ice-cold NP-40 buffer containing 50 mM Tris-HCl (pH 7.4), 300 mM NaCl, 0.5% NP-40 and protease inhibitor cocktail (Biotool) with rotation at 4°C for 45 min. Cell lysate was centrifuged at 13,000 rpm for 15 min at 4°C. The supernatant was removed into a new tube and incubated with Flag beads (Sigma, F1804, clone M2) for 3 h at 4°C, or with indicated antibody overnight followed by incubation with Protein-A beads (GE) for another 1 h at 4°C. Beads were washed three times with ice-cold NP-40 buffer, and the proteins were denatured by SDS loading buffer containing dithiothreitol.

Western blotting was performed after loading the samples into 8% SDS PAGE gel and running for about 1 h at 130 V. Subsequently, the separated proteins were transferred onto a nitrocellulose membrane. After blocked with 5% fat-free milk (BD Biosciences), the membrane was incubated with primary antibody overnight at 4°C and secondary antibody for 1 h at room temperature (RT). Then the membrane was washed 3 times with TBST and analyzed by Typhoon FLA 9500 (GE Healthcare) image scanning. Immunoblotting intensity was quantified using Image Quant TL software (GE Healthcare). The antibodies used were listed in Supplementary Table 2.

### Immunofluorescence Staining

Cells seeded in 12-well plates were washed with ice-cold PBS and fixed immediately with 4% paraformaldehyde for 30 min at room temperature (RT). Then, fixed cells were permeabilized with 0.2% Triton X-100 in phosphate-buffered saline (PBS) for 10 min at RT. Subsequently, cells were blocked in 5% bovine serum albumin (in PBS) for 60 min at RT, followed by incubation with primary antibodies against Flag/HA/Myc overnight at 4°C. After washing cells with PBS 3 times, Alex Fluor 488 (Green)/Fluor 592 (Red) conjugated secondary antibodies (Invitrogen) were added to cells for 1 h at RT. Cell nuclei were stained with DAPI (Invitrogen). Images were captured using Leica fluorescence optical microscope.

### RNA Sequencing

Stable cells were harvested with TRIzol reagent (Invitrogen) and kept at −80°C. RNA extraction and sequencing were performed by Shanghai Majorbio. Each experiment contains three biological replicates.

### Quantitative RT-PCR

Total RNA was isolated using TRIzol reagent (Invitrogen) following the manufacturer’s instruction. cDNA was synthesized using oligo-dT primers in TransScript First-Strand cDNA Synthesis SuperMix (Transgene Biotech). Real-time PCR was performed using gene-specific primers in the presence of SYBR Premix Ex Taq (TaKaRa) in a 7900HT Sequence Detection System (Applied Biosystems). β-actin was used as a housekeeping control. Primer sequences were present in Supplementary Table 2.

### Chromatin Immunoprecipitation (ChIP)-qPCR Assays

ChIP-qPCR assays were performed as previously described (Lan et al., 2007). Briefly, cells were cross-linked with 1% paraformaldehyde for 10 min at room temperature (RT), and immediately quenched by adding 0.125 M glycine. Collect cell pellet and shear the chromatin into 0.2∼1 kb fragment with sonication at 4°C for 25 min (Bioruptor, 90% power, 15 s on, 45 s off). Then, chromatin was immunoprecipitated at 4°C for 3 h with the antibody against Flag (Sigma), RUNX1 (Abcam), or mouse IgG (CST). Antibody-chromatin complexes were pulled-down using protein A beads (GE) for 1 h at 4°C with rotation. Then the beads with specific binding DNA was washed 3 times with high salt buffer (50 mM HEPE NaOH pH 7.5, 500 mM NaCl, 1 mM EDTA, 0.1% Na-Deoxycholate, 1% TritonX100), 2 times with low salt buffer (10 mM TrisCl PH8.0, 250 mM LiCl, 1 mM EDTA, 0.5% NP40, 0.5% Na-Deoxycholate), 1 time with TE buffer. After cross-link reversal and proteinase K (TaKaRa) treatment, DNA fragments were purified with PCR Purification Kit (QIAGEN) and further analyzed by real-time quantitative PCR using the primers as listed in Supplementary Table 2.

### MeDIP-qPCR Analysis

Genomic DNA from cells was prepared using a phenol-chloroform method. The MeDIP assay was performed as previously described (Ito et al., 2010). Briefly, 2 μg of genomic DNA was denatured and immunoprecipitated with the antibody against 5 mC or mouse IgG (CST) followed by pull-down with protein A-beads (GE). Beads were washed for three times and treated with proteinase K for 4 h at 65°C. DNA was purified with PCR Purification Kit (QIAGEN) and the isolated DNA was analyzed by real-time quantitative PCR using the primers as listed in Supplementary Table 2.

### Quantification and Statistical Analysis

Statistical analyses were performed using one-way or two way analysis of variance (ANOVA) with Bonferroni’s test. All data shown represent the results obtained from triplicated independent experiments with standard errors of the mean (mean ± SD). The values of *p* < 0.05 were considered statistically significant.

## Results

### Mutually Exclusive Mutational Patterns of Epigenetic Modifiers

Mutations of genes that are functionally linked in a common pathway or biological process tend not to occur concurrently in the same cell and often exhibit a pattern of mutual exclusivity. For instance, BRAF mutations in colorectal cancers occur only in tumors that do not carry mutations in RAS because both genes involved in the common RAS/RAF/MAPK signaling pathway (Rajagopalan et al., 2002). Similarly, mutations of IDH, TET2, or WT1 also occur mutually exclusively with one another as these three genes act in the same pathway. With large-scale tumor sequencing data, it is possible to identify novel functional link between genes that function in the same pathway or cellular process.

**FIGURE 1:**
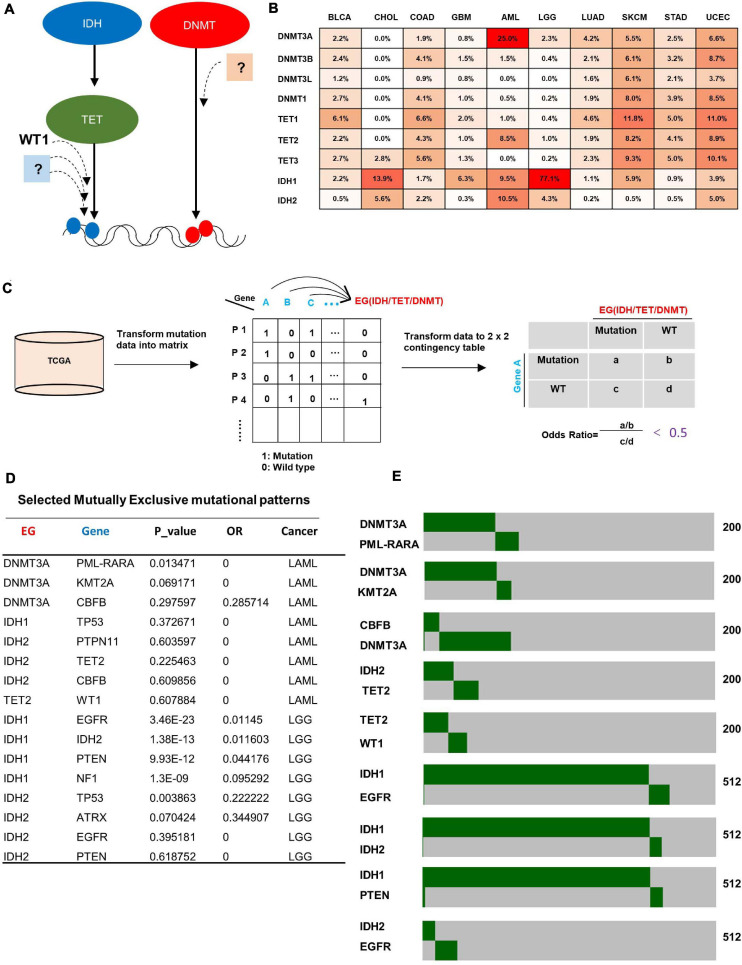
Bioinformatic search for genes mutated mutually exclusively with genes known to be involved in DNA de/methylation. **(A)** Illustration of the rationale for identifying genes that may function in the same pathway with epigenetic genes. **(B)** Mutation rate of epigenetic genes across 10 cancer types. Data was obtained from cBioportal. **(C)** Schematic diagram of methodology for identifying genes that show mutual exclusivity with mutations targeting IDH, TET, and DNMT family genes. **(D)** Selected mutually exclusive mutation pairs in AML and LGG. **(E)** Heatmap showing the mutually exclusive mutational pattern of gene pairs. The number of patients for each analysis is indicated on the right.

To identify new gene(s) that is potentially involved in the regulation of DNA de/methylation process (Figure 1A), we performed a comprehensive analysis of mutations targeting three families of genes which are known to be involved in DNA de/methylation; two IDH genes (*IDH1* and *IDH2*), three TET genes (*TET1*, *TET2*, and *TET3*) and four DNMT genes (*DNMT3A*, *DNMT3B*, *DNMT3L*, and *DNMT1*) in 34 cancer types. We found that these three families of genes were mainly mutated in 10 cancer types; bladder urothelial carcinoma (BLCA), cholangiocarcinoma (CHOL), colon adenocarcinoma (COAD), glioblastoma multiforme (GBM), acute myeloid leukemia (AML), brain lower grade glioma (LGG), lung adenocarcinoma (LUAD), skin cutaneous melanoma (SKCM), stomach adenocarcinoma (STAD), and uterine corpus endometrial carcinoma (UCEC) (Figure 1B). These findings suggest that altered DNA de/methylation may more profoundly impact the tumorigenesis of these 10 types of cancer. We subsequently focused our search for novel gene(s) involved in DNA de/methylation in these 10 cancer types.

For each of 10 cancer types, significantly mutated genes (mutation rate higher than 4%) were collected from cBioportal (Figure 1C, left), then the mutation data were transformed into a binary matrix (Figure 1C, middle), where rows represent patients while columns represent genes, and “1”/“0” denote if the gene is wild type or mutant, respectively. A 2 × 2 contingency table was generated for each gene with an epigenetic gene (IDH/TET/DNMT) (Figure 1C, right). Fisher exact test between all pairs of genes was carried out to get the *p*-value, and odd ratio was used to indicate exclusivity or co-occurrence. The gene pairs with an odds ratio below 0.5 (strong tendency toward mutual exclusivity) were selected for further analysis. A total of 1,378 gene pairs that show mutually exclusive mutation pattern were identified across the 10 cancer types (Supplementary Table 1). Confirming previous reports, we found mutual exclusive mutations between IDH1 and IDH2 (Yan et al., 2009), between IDH2 and TET2 (Gaidzik et al., 2012), between TET2 and WT1 (Wang et al., 2015), and between IDH1-EGFR (Agnihotri et al., 2014) in AML and LGG (Figures 1D,E). Notably, we also identified additional new genes which are mutated in AML in a statistically significant, mutually exclusive manner with genes know to be involved in DNA de/methylation, such as gene pairs of CBFB/DNMT3A, KMT2A/DNMT3A, PTPN11/IDH2, and ATRX/IDH2 (Figures 1D,E), suggesting potential function of these genes in regulating DNA de/methylation process.

### CBFB-MYH11 AML Patients Have a Similar DNA Hypomethylation Signature as DNMT3A Mutant Samples

**FIGURE 2:**
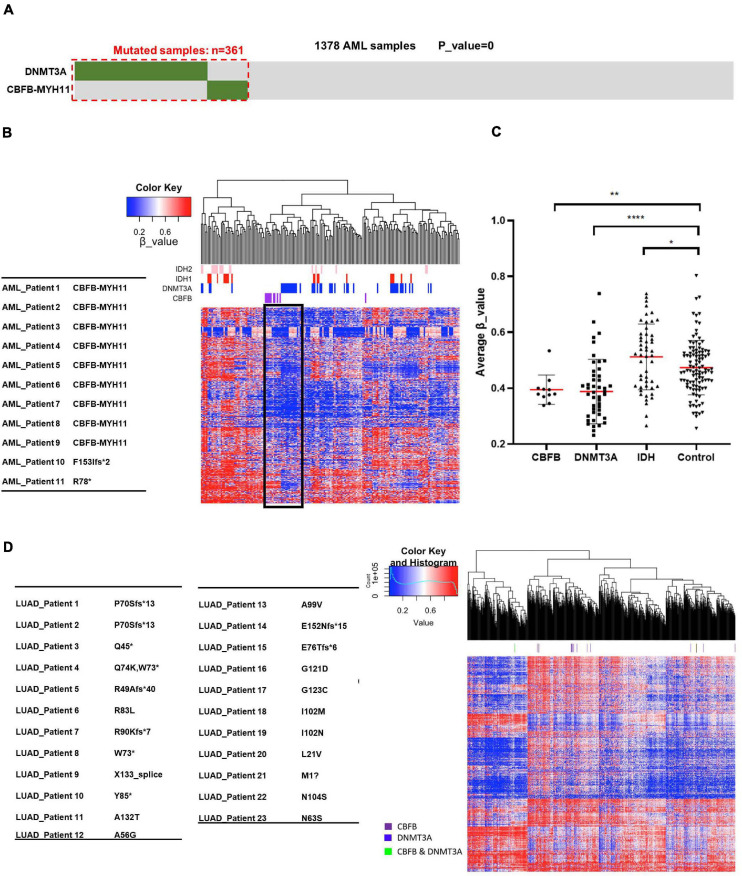
AML samples bearing CBFB-MYH11 mutations have a similar DNA hypomethylation signature as DNMT3A mutant tumors. **(A)** Mutually exclusive mutation pattern of DNMT3A and CBFB-MYH11 seen in the expanded tumor samples. Somatic variants in *DNMT3A* and *CBFB-MYH11* were identified from a total of 1,378 AML cases, in which 361 cases carried at least one mutation. **(B)** Heatmap and hierarchical clustering of AML patients based on DNA methylation. Level3 DNA methylation data of AML samples was download from TCGA database. Unsupervised hierarchical clustering was performed with hclust function with R (heatmap.3 package) using the most variably methylated CpG sites (*n* = 8,030, SD > 0.27). β_value was used to indicate the methylation level of each CpG site. **(C)** Methylation level of AML patients with different genomic alterations. Each point is one patient. Average β_value was calculated with data from **(B)**. Asterisks denote statistical significance with one-way ANOVA. *p < 0.05; **p < 0.01; ****p < 0.0001 for the indicated comparison. **(D)** Heatmap and hierarchical clustering of LUAD patients based on DNA methylation.

Among these novel mutually exclusive mutational patterns, we were intrigued by the gene pair of CBFB/DNMT3A as CBFB fusion occurs frequently in AML, which is also observed in other studies (Papaemmanuil et al., 2016; Eisfeld et al., 2017). To confirm the mutually exclusive pattern, we performed a meta-analysis of a total of 1,378 AML cases collected from published studies (Chou et al., 2010; Ley et al., 2010; Schnittger et al., 2010; Ribeiro et al., 2012; Cancer Genome Atlas Research Network et al., 2013). This analysis identified a total of 277 AML samples with DNMT3A mutation and 85 samples with CBFB mutation, including 83 samples with CBFB-MYH11 fusion. Strikingly, co-mutation of both DNMT3A and CBFB was found in only one sample (*p* = 0) (Figure 2A), providing additional evidence that CBFB mutation, especially CBFB-MYH11 fusion, occur in a mutually exclusive manner with mutations targeting DNMT3A.

To provide independent supports for the potential function in regulating DNA demethylation by CBFB, we analyzed 450K DNA methylation data set from AML samples with mutation targeting IDH, DNMT3A and CBFB. Notably, AML samples with CBFB mutation, mainly CBFB-MYH11 fusion gene mutation, were clustered together with those with mutation targeting DNMT3A (Figure 2B and Supplementary Figure 1), suggesting the possibility that mutations targeting CBFB, like those targeting DNMT3A, may impair DNA methylation process and reduce DNA methylation level.

We also performed quantitative analysis of methylation levels in groups with mutations of CBFB, DNMT3A or IDH. As previously reported (Figueroa et al., 2010; Russler-Germain et al., 2014), DNMT3A mutation and IDH mutation correlated with hypomethylation and hypermethylation signature, respectively. It is notable that similar to DNMT3A mutation, CBFB mutation resulted in a hypomethylation signature in comparison to control group (Figure 2C). These phenotypes indicated that mutations targeting CBFB, like those targeting DNMT3A, might impair DNA methylation process and reduce DNA methylation.

CBFB gene is also mutated in lung adenocarcinoma, nearly all are point mutations, but surprisingly mutations are not associated with DNA hypomethylation (Figure 2D). The result suggests the possibility that not all CBFB mutations are linked to DNA methylation impairment and the CBFB-MYH11 mutations, which are the predominant form for CBFB mutation in AML, may uniquely impact DNA methylation.

### CBFB-MYH11 Expression Regulates Genes Similar to DNMT3A Inhibition

**FIGURE 3:**
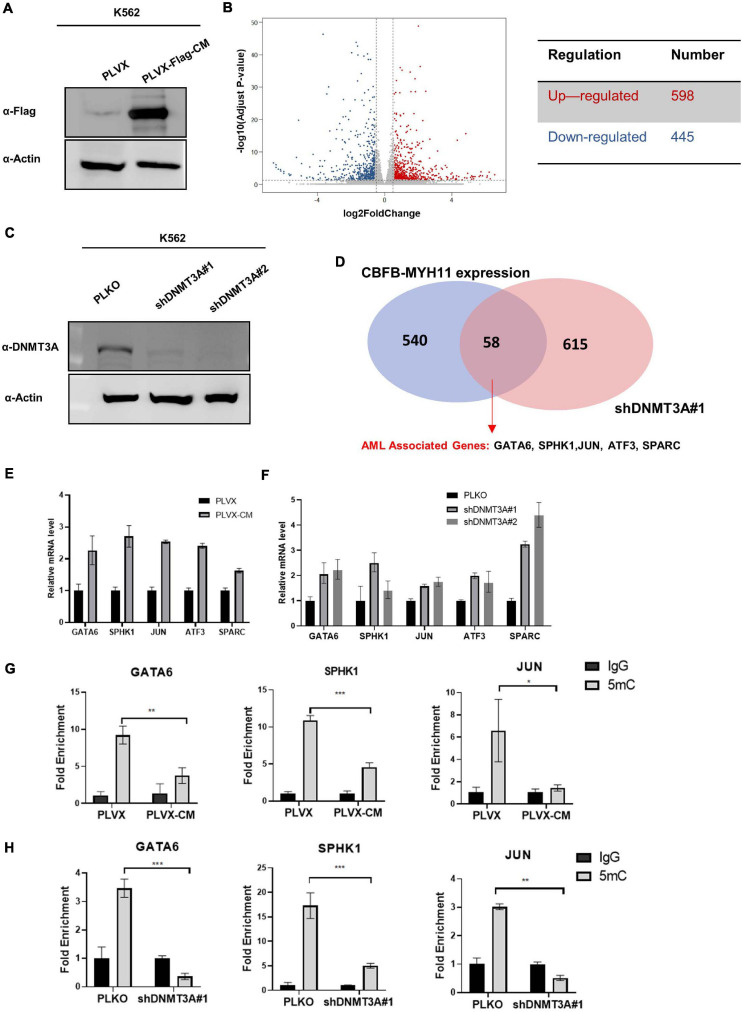
CBFB-MYH11 expression or DNMT3A inhibition regulates a common set of target genes. **(A)** Expression of Flag-tagged CBFB-MYH11 fusion protein in K562 cells. K562 cells were infected with lentivirus expressing either empty pLVX vector or Flag-tagged CBFB-MYH11 fusion protein (pLVX-Flag-CM). **(B)** Volcano plot of the differentially expressed genes between CBFB-MYH11 expressing K562 cells vs. control cells. Significantly down-regulated genes are in dark blue (sig = True), while significantly up-regulated genes are in red (sig = True), non-significant genes are in gray (sig = False). Gray vertical lines highlight log fold changes of –0.585 and 0.585. **(C)** Validation of DNMT3A knocking-down in K562 cells. K562 cells were infected by lentivirus expressing empty pLKO vector or shRNA against *DNMT3A*. **(D)** Overlap of upregulated genes in K562 cells expressing CBFB-MYH11 fusion protein or with DNMT3A knocking-down identified by RNA-seq. **(E,F)** RT-PCR confirmed that AML associated genes were up-regulated in both CBFB-MYH11 expressing cells **(E)** and DNMT3A knocking-down cells **(F)**. Data were normalized by the mRNA expression level in control cells. **(G,H)** 5 mC level of GATA6, SPHK1, JUN in K562 cells were reduced by CBFB-MYH11 expression **(H)** or DNMT3A knocking-down **(I)**. 5 mC level was determined by MeDIP-qPCR. Mouse IgG was included as negative control. Data are shown as mean ± SEM from experiments performed in triplicate. Asterisks denote statistical significance with two-way ANOVA. *p < 0.05; **p < 0.01; ***p < 0.001.

The patterns of mutually exclusive mutation and the co-clustering of hypomethylation between CBFB-MYH11 and DNMT3A led us to test directly whether CBFB-MYH11 impairs DNA methylation and gene expression. To this end, we generated K562 immortalized human myeloid leukemia cells stably expressing the empty vector control or CBFB-MYH11 fusing protein and performed RNA-seq for both cells (Figure 3A). A total of 445 genes were down-regulated by at least 1.5-fold in K562 CBFB-MYH11 cells compared to control cells. It is a bit surprising that 598 genes were found to be up-regulated by at least 1.5-fold (Figure 3B). This indicates that besides its commonly recognized function in promoting gene expression, CBFB as a co-factor of RUNX1 also suppresses the expression of a decent groups of genes.

To investigate whether the altered genes were also regulated by DNMT3A, we generated K562 cells with DNMT3A knocking-down and performed RNA-seq analysis (Figure 3C). Knocking-down of DNMT3A led to up-regulation of 673 genes by at least 1.5-fold, whose expression are likely to be suppressed by DNMT3A mediated hypermethylation. Notably, an overlap of 58 up-regulated genes were identified between CBFB-MYH11 expression and DNMT3A knocking-down cells (Figure 3D). The upregulated genes included those involved in AML progression (GATA6, SPHK1, JUN, ATF3, SPARC) (Nian et al., 2014; Powell et al., 2017; Zhou et al., 2017; Kamijo et al., 2018), and their upregulation was confirmed by RT-PCR (Figures 3E,F). We chose three of the genes (GATA6, SPHK1 and JUN) for MeDIP-qPCR analysis, which measures locus-specific DNA methylation status. Consistent with gene up-regulation, these three genes exhibit significant hypomethylation in both CBFB-MYH11 expression (Figure 3G) and DNMT3A knocking-down cells (Figure 3H). Collectively, our data suggest a possible role of DNMT3A in the epigenetic regulation of CBFB-MYH11 downstream genes.

### Inactivation of RUNX1 by CBFB-MYH11 Impairs the DNMT3A-Dependent Methylation of RUNX1 Target Genes

**FIGURE 4:**
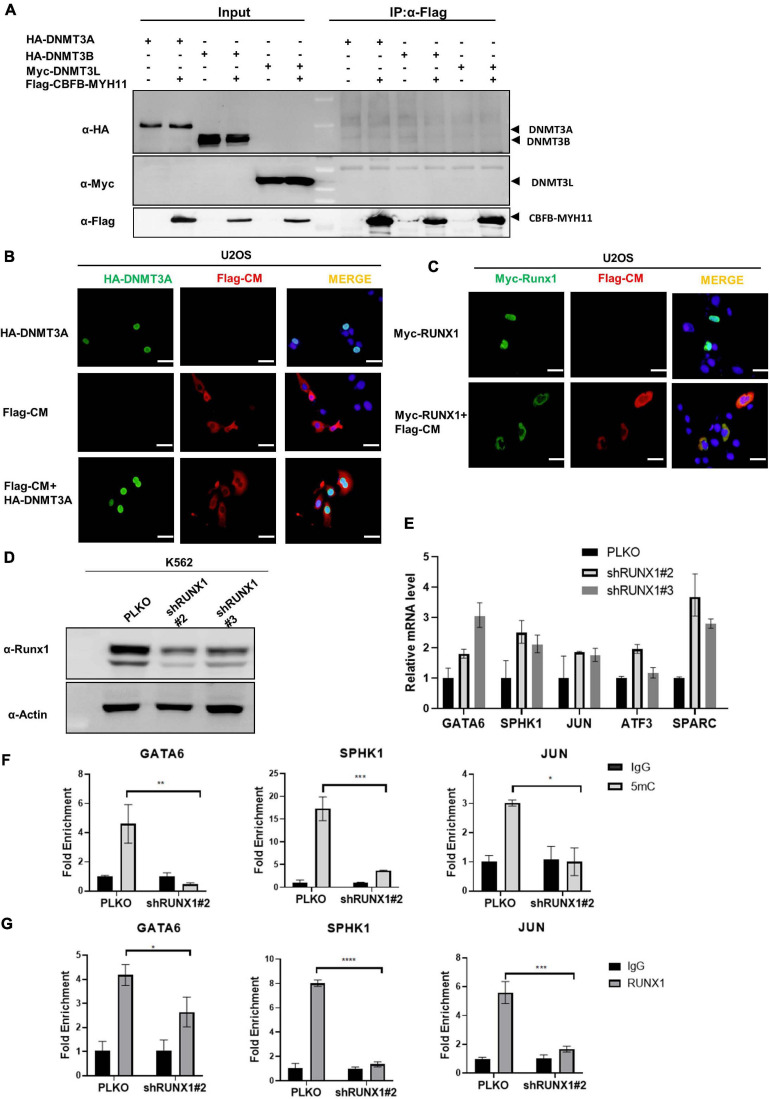
CBFB-MYH11 impairs the methylation of RUNX1 target genes. **(A)** CBFB-MYH11 does not interact with DNMT3A, DNMT3B, or DNMT3L. Plasmids expressing the indicated proteins were transiently transfected in HEK293T cells. CBFB-MYH11 fusion protein was purified by immunoprecipitation with Flag beads, followed by Western blot to detect the interaction with DNMT3A, 3B, or 3L with antibodies against HA or Myc. **(B)** CBFB-MYH11 and DNMT3A were located in different subcellular compartments. Flag-tagged CBFB-MYH11 and/or HA-tagged DNMT3A were transfected into U2OS cells. After 36 h, cells were fixed with 4% paraformaldehyde followed by immunofluorescence staining with indicated antibodies. DAPI (blue) was used for DNA staining. Scale bars: 50 μm. **(C)** RUNX1 was directed to cytoplasm by CBFB-MYH11 fusion protein. Flag-tagged CBFB-MYH11 and/or Myc-tagged RUNX1 were transfected into U2OS cells. Immunofluorescence was performed as in **(B)**. Scale bars: 50 μm. **(D,E)** AML associated genes were up-regulated in RUNX1 deficient cells. RUNX1 was knocked down by shRNAs in K562 cells. Expression of AML associated genes was determined by RT-PCR. Data were normalized by the mRNA expression level in control cells. **(F)** 5 mC level of GATA6, SPHK1, and JUN genes in K562 cells was reduced by RUNX1 knocking-down. 5 mC level was determined by MeDIP-qPCR. Mouse IgG was included as a negative control. **(G)** RUNX1 enrichment at GATA6, SPHK1 and JUN determined by ChIP-qPCR. Rabbit IgG was induced as a negative control. Data are shown as mean ± SEM from experiments performed in triplicate. Asterisks denote statistical significance with two-way ANOVA. *p < 0.05; **p < 0.01; ***p < 0.001; ****p < 0.0001 for the indicated comparison.

To determine the mechanism by which CBFB-MYH11 fusion associates with DNA hypomethylation and alters gene expression, we first tested the model that CBFB-MYH11 directly interacts DNMT3A and recruits it to target genes. However, no interaction between CBFB-MYH11 and DNMT3A, DNMT3B, or 3L was detected by co-immunoprecipitation (co-IP) (Figure 4A). This was further validated by immunofluorescence staining, which showed that the CBFB-MYH11 fusing protein was chiefly localized in the cytoplasm, consistent with a previous report (Adya et al., 1998), while DNMT3A was predominantly localized in the nucleus and not affected by CBFB-MYH11 expression (Figure 4B). These results exclude the possibility that CBFB-MYH11 oncogenic fusion physically interacts with DNMT3A and sequesters it in the cytoplasm, thereby impairing nuclear DNA methylation.

CBFB-MYH11 mutation was reported to suppress RUNX1 function through directing RUNX1 to cytoplasm (Adya et al., 1998). In line with previous work, we found that ectopically expressed RUNX1 localized in the nucleus when expressed alone but in the cytoplasm when co-expressed with CBFB-MYH11 (Figure 4C). This observation led us to investigate the possibility that RUNX1 may involve DNMT3A to regulate its target gene expression and this regulation is disrupted by the CBFB-MYH11 fusion in AML. We generated K562 stable cells with RUNX1 knocking-down and found that the AML associated genes mentioned above were also up-regulated (Figures 4D,E). In addition, MeDIP-qPCR data showed that GATA6, SPHK1, and JUN were hypomethylated in RUNX1 knocking-down cells (Figure 4F). These data are consistent with observations in CBFB-MYH11 expression and DNMT3A knocking-down cells (Figure 3H). Chromatin immunoprecipitation (ChIP) assay showed that RUNX1 directly bound to these genes (Figure 4G), consistent with data from previous RUNX1 ChIP-seq (Prange et al., 2017). Together, these findings support a model that CBFB-MYH11 fusion protein sequesters RUNX1 in the cytoplasm, resulting in its functional inactivation.

### RUNX1 Directly Interacts With DNMT3A

**FIGURE 5:**
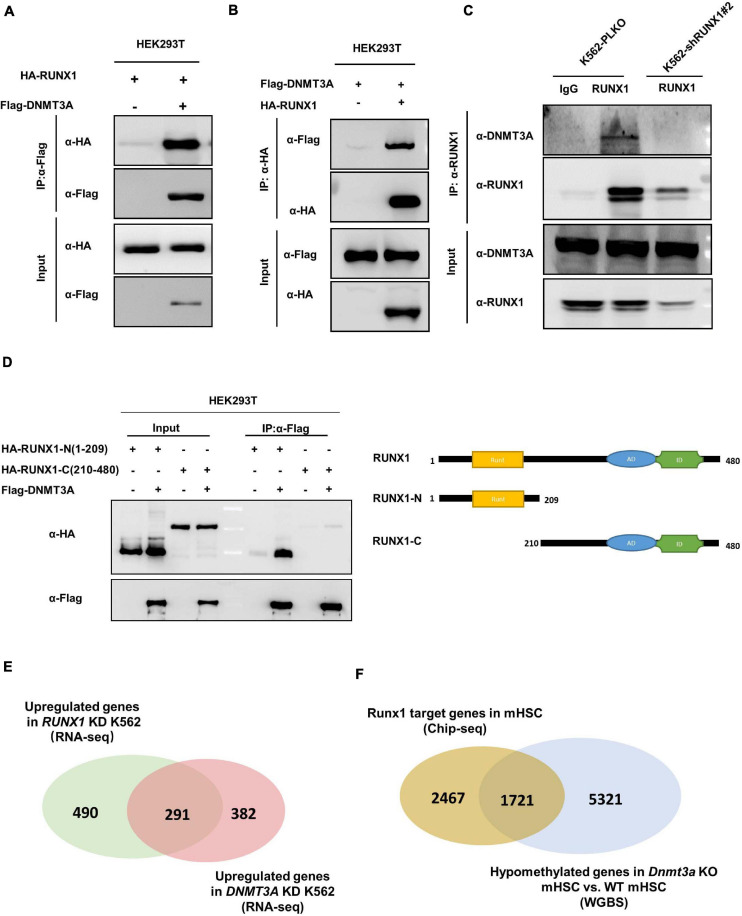
RUNX1 directly interacts with DNMT3A. **(A,B)** DNMT3A interacts with RUNX1 when exogenously expressed in HEK293T cells. The interaction was detected in either immunoprecipitation of DNMT3A **(A)** or reciprocal immunoprecipitation of RUNX1 **(B)**. **(C)** Endogenous DNMT3A and RUNX1 interact with each other. Endogenous RUNX1 protein in K562 cells was immunoprecipitated and DNMT3A was detected with the specific antibody. **(D)** DNMT3A interacts with the N-terminal domain of RUNX1. Flag-tagged DNMT3A was co-expressed with RUNX1 full-length or truncations in HEK293T cells. DNMT3A was immunoprecipitated followed by Western blot to detect HA-RUNX1s. **(E)** Venn diagrams depicting overlap of upregulated genes from RNA-Seq (Fold change > 1.5) in K562 cells with RUNX1 or DNMT3A knocking-down. **(F)** Venn diagrams depicting overlap between RUNX1 target genes identified by ChIP-seq in mouse hematopoietic stem cells and hypomethylated genes in mHSC with *Dnmt3a* knocking out.

Reduced DNA methylation by the RUNX1 knockdown led us to test whether it physically interacts with DNMT3A and thus recruits DNMT3A to target genes. We found, in fact, that the DNMT3A-RUNX1 association can be readily detected by co-IP assay when both proteins were ectopically expressed (Figures 5A,B). Moreover, endogenous interaction of the two proteins was also detected in K562 cells (Figure 5C). We next mapped the domain(s) in RUNX1 protein that mediates the interaction, and found the N-terminus of RUNX1 (residues 1–209) containing the Runt domain is required for its interaction with DNMT3A (Figure 5D). The above observations suggest that RUNX1 may recruit DNMT3A to modulate DNA methylation and expression of target genes.

To provide functional evidence supporting RUNX1-DNMT3A association, we preformed RNA-Seq in RUNX1 knocking-down K562 cells. Notably, we found that nearly 37.3% of upregulated genes (Fold change > 1.5) in RUNX1 knocking-down cells overlap with DNMT3A target genes (Figure 5E). To further corroborate this finding, we analyzed the data from Runx1 ChIP-seq performed in mouse hematopoietic stem cells (mHSC) (Lam et al., 2014) and data from whole genome bisulfite sequencing (WGBS) in *Dnmt3a* knockout mHSC (Challen et al., 2014). Comparison of these two sets of data demonstrated a significant overlap (*p* < 0.001) between Runx1 target genes (41.1%) and hypomethylated genes induced by *Dnmt3A* knockout (24.1%) (Figure 5F). Collectively, these results raise a model that RUNX1 and DNMT3A function in a common pathway.

### CBFB-MYH11 Mutation Disrupts the Interaction Between DNMT3A and RUNX1

**FIGURE 6:**
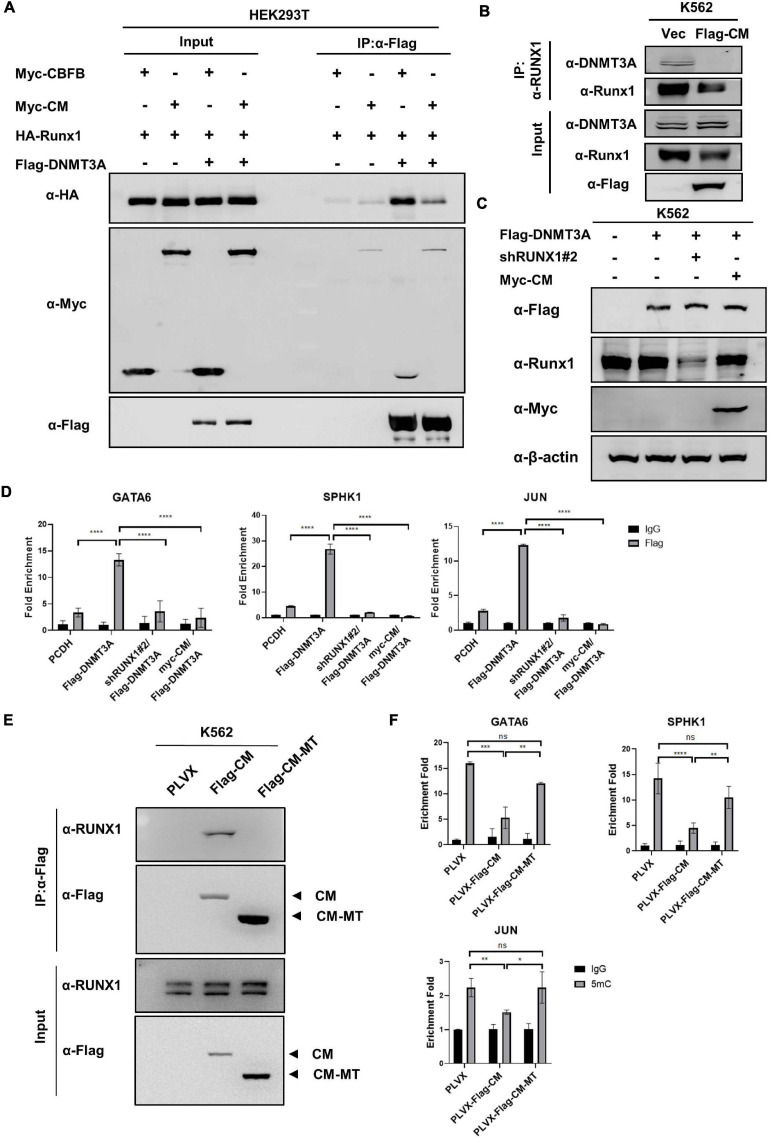
CBFB-MYH11 mutation disrupts the interaction between DNMT3A and RUNX1. **(A)** CBFB-MYH11 fusion protein disrupted the interaction between DNMT3A and RUNX1. The indicated proteins were expressed in HEK293T cells. DNMT3A was purified by immunoprecipitation with Flag beads, followed by Western blot with antibodies against HA or Myc. **(B)** The interaction between DNMT3A and RUNX1 was impaired in CBFB-MYH11 expressing cells. Endogenous RUNX1 protein in K562 cells or CBFB-MYH11 expressing cells was immunoprecipitated and DNMT3A was detected with the specific antibody. **(C)** Expression of CBFB-MYH11 fusion protein or knockdown of RUNX1 in K562 cells stably expressing DNMT3A. Protein expression level was determined by Western blot. **(D)** DNMT3A binding to GATA6, SPHK1 and JUN genes was hindered by either RUNX1 knocking-down or CBFB-MYH11. The occupancy of DNMT3A on target genes was determined by ChIP-qPCR. Rabbit IgG was used as a negative control. Data are shown as mean ± SEM from experiments performed in triplicate. Asterisks denote statistical significance with two-way ANOVA. ****p < 0.0001 for the indicated comparison. **(E)** CBFB-MYH11-MT does not interact with RUNX1. Flag tagged CBFB-MYH11 (Flag-CM) or CBFB-MYH11-MT (Flag-CM-MT) was immunoprecipitated with Flag beads and RUNX1 binding was detected with antibody against RUNX1. **(F)** CBFB-MYH11 relies on RUNX1 to regulate DNA methylation. 5 mC level was determined by MeDIP-qPCR. Mouse IgG was included as a negative control. Data are shown as mean ± SEM from experiments performed in triplicate. Asterisks denote statistical significance with two-way ANOVA. *p < 0.05; **p < 0.01; ***p < 0.001.

Above findings led us to hypothesize that CBFB-MYH11 fusion protein may interfere the interaction between RUNX1 and DNMT3A by sequestering RUNX1 in the cytoplasm. This hypothesis was supported by the observation that expression of CBFB-MYH11 significantly reduced the interaction between DNMT3A and RUNX1 (Figures 6A,B). We speculated that DNMT3A was recruited by RUNX1 to suppress certain genes, while this effect was disrupted by CBFB-MYH11. To test this hypothesis, we knocked down RUNX1 or overexpressed CBFB-MYH11 in cells stably expressing DNMT3A (Figure 6C). ChIP-qPCR was performed by immunoprecipitation of Flag-tagged DNMT3A. The data showed that DNMT3A could bind to GATA6, SPHK1, and JUN genes in control cells, while either RUNX1 knocking-down or CBFB-MYH11 expression similarly blocked DNMT3A enrichment on these genes (Figure 6D).

To get further evidence that CBFB-MYH11 relies on RUNX1 to regulate DNA methylation, we expressed a RUNX1 binding-deficient CBFB-MYH11 (CBFB-MYH11-MT), in which the N-terminal 1–135 amino acids of CBFB-MYH11was deleted (Speck and Gilliland, 2002), in K562 cells. The defect of CBFB-MYH11-MT in binding to RUNX1 was confirmed by Co-IP (Figure 6E). Subsequent ChIP-qPCR results showed although CBFB-MYH11 expression caused DNA hypomethylation of GATA6, SPHK1, and JUN genes, CBFB-MYH11-MT is largely defective in inducing DNA hypomethylation in these genes (Figure 6F), indicating RUNX1 binding is required for CBFB-MYH11 induced DNA hypomethylation.

**FIGURE 7:**
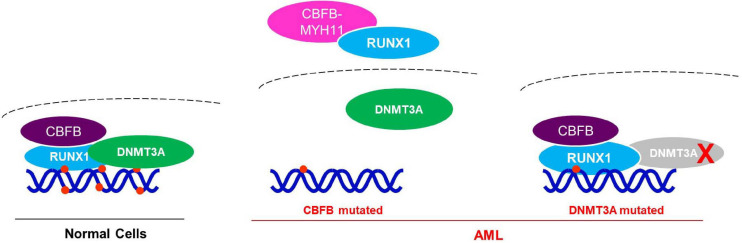
Working model for the potential mechanism of CBFB-MYH11 in regulating RUNX1 target genes. In normal cells, DNMT3A is recruited by RUNX1/CBFB to methylate and suppress target genes. While in the CBFB-MYH11 mutant cells, RUNX1 is cytoplasmic and unable to recruit DNMT3A to target genes, leading to hypomethylation and upregulation of target genes. Alternatively, in tumor cells bearing DNMT3A catalytic mutant, the mutant DNMT3A fails to methylate RUNX1 target genes.

Collectively, these results support a model that RUNX1 recruits DNMT3A to target genes, while CBFB-MYH11 mutation disrupts the interaction between DNMT3A and RUNX1. Therefore, CBFB-MYH1 may up-regulate some AML associated genes by acting through RNUX1-DNMT3A to reduce DNA methylation of the genes (Figure 7).

## Discussion

Growing evidences link the aberrant DNA methylation with tumorigenesis. Taking the advantages of massive cancer genomic sequencing, we attempted to identify novel genes involved in the regulation of DNA de/methylation by using bioinformatic analysis of mutation patterns of genes with genes known to be involved DNA de/methylation process. Our study identified several genes that are mutated in mutually exclusive pattern with genes of *DNMT*, *TET*, and *IDH* families in different tumors. We further validate the genetic link of identified genes with genomic DNA methylation data which provide an independent and functional support. We believe that this approach is generally applicable to the search for other novel genetic interaction using the continuingly expanding publicly available cancer genomic data. The findings from these bioinformatic studies may guide for mechanistical investigations and uncover potential therapeutic opportunities as we have demonstrated in this study with the discovery of mutually exclusive mutations targeting DNMT3A and CBFB-MYH11 fusion in AML.

To the best of our knowledge, this is the first report that links the function of RUNX1 transcription factor to the recruitment of DNMT3A and provide the first explanation on how CBFB-MYH11 fusion is associated with DNA hypomethylation. Functionally, we showed that expression of CBFB-MYH11 fusion protein, like knockdown of DNMT3A, significantly up-regulated a common group of genes in K562 cells, some of which are involved in AML progression. In line with their increased expression, DNA methylation of these AML-related genes are reduced. It was previously reported that the oncogene *MN1* was hypomethylated and overexpressed in CBFB-MYH11 mutant AML patients, partially due to the decreased expression of DNMT3B (Larmonie et al., 2018). But how DNMT3B down-regulation and MN1 hypomethylation is linked to CBFB-MYH11 mutation has not been elucidated. Our work reveals a mechanism for CBFB-MYH11 in DNA methylation control by targeting DNMT3A via RUNX1, and thus providing possible molecular basis for CBFB-MYH11 in leukemogenesis.

Although bioinformatic analysis identified the significant association between CBFB-MYH11 fusion and DNMT3A mutation, the direct protein interaction is between DNMT3A and RUNX1 instead of CBFB. This is different from the mutations targeting WT1 which interacts with TET2 directly (Wang et al., 2015). Our findings provide an interesting example where an oncogenic event indirectly targets the DNA de/methylation enzyme through altering the function of another protein. Previous work has shown that RUNX1 proteins can bind and recruit a range of co-activators such as MYB, p300, ETS, SMADs and co-repressors such as HDACs, mSin3A, nCoR, SUV39H1 and thereby serve as orchestrators of transcription at target promoters (Blyth et al., 2005; Reed-Inderbitzin et al., 2006). We speculate that the cytoplasmic sequestration of RUNX1 by the CBFB-MYH11 fusion could also impair the function RUNX1 with these additional transcriptional regulators and histone modifying enzymes. Interestingly, another fusion gene RUNX1-RUNX1T1 is also frequently mutated in AML, and show mutually exclusive pattern with DNMT3A mutations (Papaemmanuil et al., 2016; Eisfeld et al., 2017). However, since RUNX1-RUNX1T1 fusion is localized in nucleus and may not affect RUNX1 nuclear localization, whether RUNX1-RUNX1T1 functionally links to DNMT3A need further investigation.

Our studies also bear a therapeutic implication. In clinical treatment, patients harboring CBFB-MYH11 fusion mutation, which accounts for about 6∼8% in total AML patients, are sensitive to high-dose cytarabine (ara-C) treatment. Nonetheless, large amounts of studies reported that the relapse free survival (RFS) of ara-C treatment is only 40∼60% at 3∼5 years and about 40% at 10 years, leaving great room for improvement (Borthakur et al., 2008). Our studies suggest the possibility of targeting CBFB-MYH11 fusion protein, for example by the targeted protein degradation (also known as proteolysis targeting chimera or PROTAC) technology, could release sequestered RUNX1 and restore the RUNX1-DNMT3A regulatory network. One intriguing possibility is that the restoration of the RUNX1-DNMT3A interaction may lead to re-repression of oncogenes that are normally suppressed by the DNA methylation brought by the RUNX1-DNMT3A interaction, but were activated after the cytoplasmic sequestration of RUNX1 by the CBFB-MYH11 fusion.

## Data Availability Statement

The datasets presented in this study can be found in online repositories. The names of the repository/repositories and accession number(s) can be found below: (GEO: GSE169682).

## Author Contributions

PL, YX, and H-XY conceived the general framework of this study. PL, MZ, and YL performed the bioinformatics analysis. PL, J-PL, S-JS, and H-XY designed the experiments and prepared the manuscript. PL, J-PL, S-JS, YG, YA, XC, and YS performed the experiments. All authors contributed to the article and approved the submitted version.

## Conflict of Interest

YX was employed by company Cullgen Inc. The remaining authors declare that the research was conducted in the absence of any commercial or financial relationships that could be construed as a potential conflict of interest.
