# Supplementary figures and images for: CBFB-MYH11 Fusion Sequesters RUNX1 in Cytoplasm to Prevent DNMT3A Recruitment to Target Genes in AML
Source: Front Cell Dev Biol. 2021 Jul 15;9:675424. doi: 10.3389/fcell.2021.675424 (PMC8321512; doi:10.3389/fcell.2021.675424)

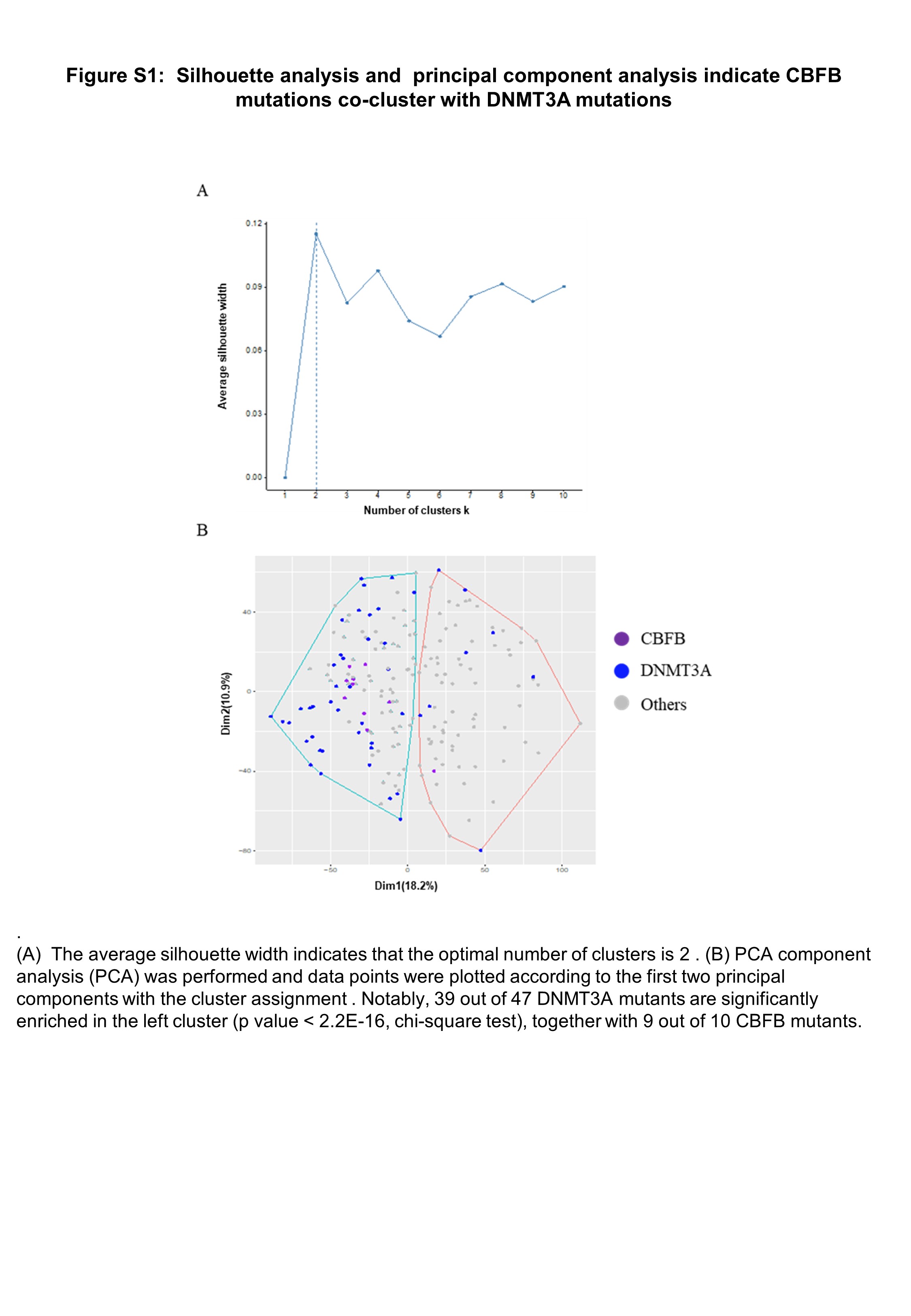

Supplement: Supplementary file 3 [file Image_1.JPEG]
